# Percutaneous image-guided lumbar decompression and outpatient laminectomy for the treatment of lumbar spinal stenosis: a 2-year Medicare claims benchmark study

**DOI:** 10.1016/j.inpm.2024.100412

**Published:** 2024-04-22

**Authors:** Peter S. Staats, Michael J. Dorsi, David E. Reece, Natalie H. Strand, Lawrence Poree, Jonathan M. Hagedorn

**Affiliations:** a National Spine and Pain Centers, Atlantic Beach, FL, USA; b UCLA, Westlake Village Primary & Specialty Care, 1250 La Venta Drive, Westlake Village, CA, 91361, USA; c Walter Reed National Military Medical Center, 8901 Wisconsin Ave, Bethesda, MD, 20889, USA; d Anesthesiology and Pain Medicine, Mayo Clinic Arizona, 5777 E. Mayo Blvd, Phoenix, AZ, 85054, USA; e Department of Anesthesia and Perioperative Care, University of California at San Francisco, UCSF Pain Management Center, 2255 Post Street, San Francisco, CA, 94115, USA; f iSpine Pain Physicians, 9645 Grove Circle N, Maple Grove, MN, 55369, USA

**Keywords:** Lumbar spinal stenosis, Neurogenic claudication, *mild*, Outpatient laminectomy, Medicare claims, Percutaneous image-guided lumbar decompression, PILD

## Abstract

**Background:**

This prospective longitudinal study compares outcomes for Medicare beneficiaries receiving outpatient percutaneous image-guided lumbar decompression (PILD) using the *mild*® procedure to patients undergoing outpatient laminectomy. All patients were diagnosed with lumbar spinal stenosis (LSS) with neurogenic claudication (NC).

**Methods:**

All medical claims for 100 % of Medicare beneficiaries were reviewed, with study subjects identified using Centers for Medicare and Medicaid Research Identifiable Files. Baseline data were extracted individually to allow for longitudinal analysis through two-year follow-up. The index procedure was defined as the first *mild* or outpatient laminectomy during the enrollment period. The rate of subsequent surgical procedures and incidence of harms were used as study outcomes.

**Results:**

Cohorts included 2197 *mild* and 7416 laminectomy patients. *mild* patients were significantly older (76.7 years versus 73.4 years, respectively; p < 0.0001), and 57.4 % of *mild* were female, compared to 43.3 % of laminectomy (p < 0.0001). *mild* patients presented with significantly more baseline comorbidities compared to laminectomy patients (mean of 5.7 versus 4.8, respectively; p < 0.0001). Subsequent surgical procedure rate of 9.0 % for *mild* was significantly higher than 5.5 % for laminectomy (p < 0.0001). *mild* experienced harms at a significantly lower rate than laminectomy (1.9 % versus 5.8 %, respectively; p < 0.0001). The composite rate of subsequent surgical procedures and harms was similar between groups at 10.8 % for *mild* and 11.0 % for laminectomy.

**Conclusions:**

*mild* can be considered a viable option for treatment of LSS with NC as evidenced by real-world data in this study. At two-years, *mild* patients experienced fewer harms and underwent more subsequent surgical procedures than laminectomy patients. The higher rate of subsequent surgical procedures for *mild* may be attributable to its position earlier in the LSS treatment algorithm. The overall rate of harms and subsequent surgical procedures was similar between groups, suggesting that *mild* should be considered as a treatment option, particularly for older patients with multiple comorbidities.

## 1 Introduction

Lumbar spinal stenosis (LSS) is a common degenerative spinal condition that most often impacts the elderly and is associated with chronic pain and reduced mobility [[1], [2], [3]]. Treatment of LSS generally begins with conservative care, followed by non-surgical minimally invasive interventions such as injections and PILD, and then surgery if symptoms persist or recur. An important tenet of treatment plans for any medical condition is that early management involves non-invasive or minimally invasive therapies with lower risk when compared to treatments that are later in the algorithm. This is especially important for patients presenting with high comorbidities or advanced age [4]. It is notable that LSS has been reported to be the most common indication for lumbar spine surgery in older adults [2]. Given the increased risks of open surgery, which is associated with longer recovery times and higher complication rates compared to less invasive procedures, the care continuum for the elderly LSS patient population must be considered [2,4,5].

The percutaneous *mild®* procedure (Vertos Medical, Aliso Viejo, CA) has been available in the US since 2006 for treatment of LSS with neurogenic claudication (NC) secondary to ligamentum flavum hypertrophy [6]. This procedure has demonstrated significant improvement in mobility and reduction in pain in multiple Level 1 randomized controlled trials (RCTs), and as a minimally invasive option, is positioned early in the LSS treatment algorithm along with epidural steroid injections [3,[7], [8], [9]].

The purpose of this population-based longitudinal study was to compare two-year rates of subsequent surgical procedures and harms for Medicare beneficiaries receiving either *mild* or outpatient laminectomy for treatment of LSS with NC using Medicare claims data. Although *mild* and surgical decompression patients may have different treatment indications, the use of claims data allows for the comparison of two procedures that treat LSS but have disparate positions on the treatment algorithm. Outpatient laminectomy was chosen as a comparator to provide a more relevant comparison to the *mild* procedure which is also outpatient. To validate study methods, outcomes from this study were compared to other published reports of two-year subsequent surgical procedure rates for LSS patients treated with laminectomy using claims data. Importantly, while this analysis focused specifically on patients treated with laminectomy in an outpatient setting, the definition of subsequent surgical procedures and methods used in this report were consistent with that used in these comparative analyses [4,[10], [11], [12], [13], [14], [15]].

## 2 Materials and methods

### 2.1 Data source

All medical claims for 100 % of Medicare beneficiaries were reviewed, with study subjects identified using the Centers for Medicare and Medicaid Services (CMS) Research Identifiable Files (RIFs). Eligibility data, baseline characteristics, subsequent surgical procedures, and harms were identified and classified using International Classification of Diseases, Tenth Edition (ICD-10) administrative codes and American Medical Association's (AMA's) Current Procedural Terminology (CPT). All baseline data, including patient history, demographics, and comorbidities were extracted on an individual basis to allow for longitudinal analysis with outcomes. The index procedure was defined as the first *mild* or outpatient laminectomy procedure for an individual patient during the enrollment period. The study protocol was reviewed by WCG institutional review board (IRB) and determined to be exempt from IRB oversight (Department of Health and Human Services regulations 45 CFR 46).

### 2.2 Patient selection

**Fig. 1:**
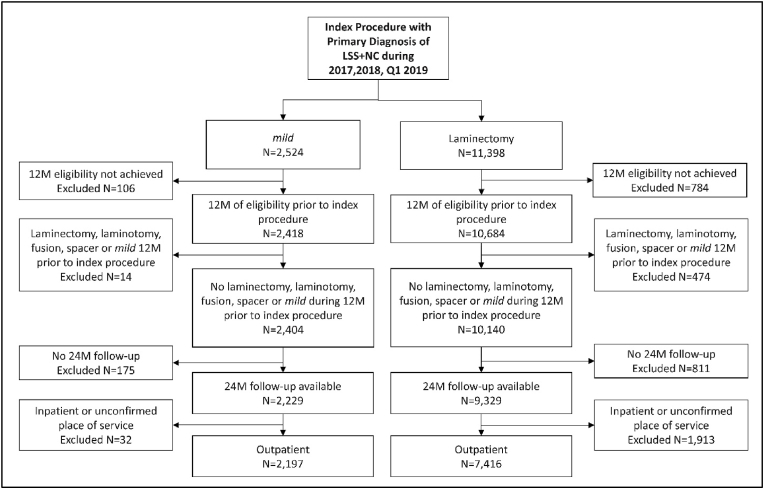
Patient selection flow chart.

All patients with a primary diagnosis of LSS with NC (ICD-10-CM diagnosis code M48.062) and treated with either a *mild* procedure (CPT code 0275T) or laminectomy (CPT code 63047) during the enrollment period from January 1, 2017 through March 31, 2019 were considered for inclusion. Patients receiving single or multi-level procedures were included. Patients were excluded if they were not enrolled in Medicare for at least 12 months prior to the index procedure, if they were treated with laminectomy, laminotomy, lumbar fusion, interspinous spacer, or *mild* during the 12 months prior to the index procedure, or if two years of follow-up data was not available. Two-year follow-up was calculated from the date of each index procedure. And finally, patients who were treated in an inpatient setting or unconfirmed place of service were excluded. Patient selection for this study is illustrated in Fig. 1.

### 2.3 Measures of comorbidity

Baseline comorbidities were identified and compared using 38 Elixhauser conditions refined for ICD-10-CM (v2022.1). In addition, three Clinical Classifications Software Refined for ICD-10-CM Diagnoses for presence of acute myocardial infarction, cardiac dysrhythmias, and fluid and electrolyte disorders were included [16] (see https://www.hcup-us.ahrq.gov/toolssoftware/ccsr/dxccsr.jsp). A search for diagnosis codes occurring during the 12 months prior to index was used to identify patients with these pre-existing comorbidities.

### 2.4 Outcomes

Rate of subsequent surgical procedures, incidence of harms, and combined rate of subsequent surgical procedures and harms during the two years following the index procedure were used as study outcomes.

### 2.5 Subsequent surgical procedures

Subsequent surgical procedures were defined as the first lumbar surgery following the index procedure. Multiple subsequent surgical procedures were counted if they occurred on the same day but only one incident was considered when calculating the overall procedure rate. The overall rate is the percent of total patients receiving at least one lumbar surgery after the index. Subsequent surgical procedures included disc procedures, endoscopic decompression, vertebral fusion, laminectomy/laminotomy, and implantation of a spacer with open decompression, and a primary diagnosis code of LSS with NC was required.

### 2.6 Harms

Harms were identified using diagnosis and procedure codes and confirmed as being associated with the index procedure. Consistent with other open decompression claims publications, all harms, except for heterotopic ossification, were restricted to inpatient claims, including those occurring on the day of index [4,13,15]. Harms categories are defined in Table 1, together with the time period for which harms would be considered. Deaths occurring more than 30 days beyond the index procedure were deemed unrelated [4,13,15].

**Table 1:** Harms categories.

Harm	Timing from index procedure
Wound problems: intraoperative or post-procedural hemorrhage, hematoma, or seroma, post-operative infection, disruption of wound, incision and drainage, wound debridement, and treatment of dehiscence	Within 90 days
Life threatening complications: acute myocardial infarction, pneumonia, respiratory problems, pulmonary embolism, and stroke	Within 30 days
Neural trauma: lumbosacral spinal cord or nerve root injury, dural tear	Within 30 days
Deep vein thrombosis (DVT)	Within 30 days
Heterotopic ossification	Within 2 years
Death	Within 30 days

### 2.7 Statistical analysis

Descriptive statistics were used for demographic and baseline comorbidity data. Comparison of demographic and baseline comorbidity information was performed using a chi-square test of independence for categorical variables and an independent-samples *t*-test for continuous variables. Propensity scoring was utilized for age at index procedure, gender, race, year of index procedure, and all baseline comorbidities since subjects were not randomized to the treatment groups. Subsequent surgical procedures and harms were analyzed using Cox proportional hazards regression adjusted for propensity score.

Significance was set at 0.05 with no adjustments for multiple testing. Subjects were censored during two-year follow-up due to death unrelated to index procedure or loss of Medicare eligibility. In compliance with regulations, all outcome counts with less than 11 patients were censored to reduce potential for subject identification. All analyses utilized SAS Enterprise Guide 7.1 (Cary, NC).

## 3 Results

### 3.1 Study population

Following application of clinical inclusion and exclusion criteria, cohorts consisted of 2197 *mild* and 7416 outpatient laminectomy patients. *mild* patients were significantly older than laminectomy (76.7 years versus 73.4 years, respectively; p < 0.0001). A significant difference was seen between groups in gender, where 57.4 % of *mild* was female, compared to 43.3 % of laminectomy (p < 0.0001). Race was also significantly different between groups (p < 0.0001). (See Table 2.).

**Table 2:** Patient demographics and baseline characteristics.

	*mild* N = 2197	Outpatient Laminectomy N = 7416	p-value
Age, years^a^			
Mean ± SD (N)	76.7 ± 7.7	73.4 ± 7.5	<0.0001
Median (Min, Max)	76.0 (47, 102)	73.0 (26, 96)	
Gender			
Female	1262 (57.4 %)	3211 (43.3 %)	<0.0001
Male	935 (42.6 %)	4205 (56.7 %)	
Race			
White	1962 (89.3 %)	6843 (92.3 %)	<0.0001
Black	137 (6.2 %)	242 (3.3 %)	
Asian	20 (0.9 %)	56 (0.8 %)	
Hispanic	22 (1.0 %)	30 (0.4 %)	
Other	30 (1.4 %)	135 (1.8 %)	
Unknown	26 (1.2 %)	110 (1.5 %)	

a Age as of index procedure date.

*mild* patients presented with a mean of 5.7 baseline comorbidities compared to 4.8 for outpatient laminectomy (p < 0.0001). Significant differences in incidence rates were found in 27 of 41 individual comorbidities, with higher rates in the *mild* group for all comorbidities with significant differences. The comprehensive list of baseline comorbidities is presented in Table 3.

**Table 3:** Baseline comorbidities.

	*mild* N = 2197	Outpatient Laminectomy N = 7416	p-value
Comorbidity Count			
Mean ± SD	5.7 ± 3.5	4.8 ± 3.1	<0.0001^b^
**Comorbidities**			
Acquired immune deficiency syndrome	a	11 (0.1 %)	a
Alcohol abuse	41 (1.9 %)	121 (1.6 %)	0.4531
Deficiency anemias	603 (27.4 %)	1487 (20.1 %)	<0.0001^b^
Autoimmune conditions	230 (10.5 %)	651 (8.8 %)	0.0159^b^
Chronic blood loss anemia	57 (2.6 %)	133 (1.8 %)	0.0178^b^
Leukemia	23 (1.0 %)	55 (0.7 %)	0.1613
Lymphoma	33 (1.5 %)	95 (1.3 %)	0.4273
Metastatic cancer	25 (1.1 %)	90 (1.2 %)	0.7744
Solid tumor without metastasis, in situ	112 (5.1 %)	265 (3.6 %)	0.0012^b^
Solid tumor without metastasis, malignant	358 (16.3 %)	1051 (14.2 %)	0.0135^b^
Cerebrovascular disease	385 (17.5 %)	1026 (13.8 %)	<0.0001^b^
Coagulopathy	131 (6.0 %)	428 (5.8 %)	0.7364
Dementia	129 (5.9 %)	247 (3.3 %)	<0.0001^b^
Depression	528 (24.0 %)	1443 (19.5 %)	<0.0001^b^
Diabetes with chronic complications	560 (25.5 %)	1641 (22.1 %)	0.0010^b^
Diabetes without chronic complications	766 (34.9 %)	2323 (31.3 %)	0.0018^b^
Drug abuse	152 (6.9 %)	240 (3.2 %)	<0.0001^b^
Heart failure	297 (13.5 %)	692 (9.3 %)	<0.0001^b^
Hypertension, complicated	549 (25.0 %)	1322 (17.8 %)	<0.0001^b^
Hypertension, uncomplicated	1895 (86.3 %)	5995 (80.8 %)	<0.0001^b^
Liver disease, mild	178 (8.1 %)	571 (7.7 %)	0.5365
Liver disease, moderate to severe	a	39 (0.5 %)	a
Chronic pulmonary disease	622 (28.3 %)	1728 (23.3 %)	<0.0001^b^
Neurological disorders affecting movement	186 (8.5 %)	499 (6.7 %)	0.0054^b^
Other neurological disorders	88 (4.0 %)	240 (3.2 %)	0.0811
Seizures and epilepsy	58 (2.6 %)	153 (2.1 %)	0.1050
Obesity	689 (31.4 %)	2170 (29.3 %)	0.0586
Paralysis	40 (1.8 %)	150 (2.0 %)	0.5502
Peripheral vascular disease	667 (30.4 %)	1914 (25.8 %)	<0.0001^b^
Psychoses	85 (3.9 %)	327 (4.4 %)	0.2719
Pulmonary circulation disease	86 (3.9 %)	198 (2.7 %)	0.0025^b^
Renal failure, moderate	440 (20.0 %)	1070 (14.4 %)	<0.0001^b^
Renal failure, severe	82 (3.7 %)	173 (2.3 %)	0.0003^b^
Hypothyroidism	658 (29.9 %)	1830 (24.7 %)	<0.0001^b^
Other thyroid disorders	158 (7.2 %)	427 (5.8 %)	0.0135^b^
Peptic ulcer with bleeding	48 (2.2 %)	157 (2.1 %)	0.8469
Valvular disease	431 (19.6 %)	1236 (16.7 %)	0.0013^b^
Weight loss	111 (5.1 %)	265 (3.6 %)	0.0017^b^
Acute myocardial infarction	57 (2.6 %)	137 (1.8 %)	0.0287^b^
Cardiac dysrhythmias	544 (24.8 %)	1646 (22.2 %)	0.0118^b^
Fluid and electrolyte disorders	392 (17.8 %)	1004 (13.5 %)	<0.0001^b^

a Amount censored due to count <11.

b Incidence was statistically significantly higher for *mild* patients.

### 3.2 Subsequent surgical procedure rate

Patients receiving *mild* experienced a 9.0 % subsequent surgical procedure rate compared to 5.5 % for outpatient laminectomy patients (p < 0.0001). The rate of disc procedures was not statistically significantly different between groups, and the count of endoscopic decompressions was less than 11 in each group so therefore the number was censored. The frequency of fusion, laminectomy/laminotomy and interspinous spacer with open decompression was significantly higher for *mild* patients (See Table 4.).

**Table 4:** Rate of subsequent surgical procedures during two-year follow-up.

First Subsequent Surgical Procedure	*mild* N = 2197		Outpatient Laminectomy N = 7416		p-value^b^
Subsequent surgical procedure rate	198	9.0 %	410	5.5 %	<0.0001^c^
Disc procedure	24	1.1 %	76	1.0 %	0.1838
Endoscopic decompression	a	a	a	a	n/a
Fusion	79	3.6 %	190	2.6 %	<0.0001^c^
Laminectomy/laminotomy	134	6.1 %	283	3.8 %	<0.0001^c^
Spacer (with open decompression)	31	1.4 %	23	0.3 %	<0.0001^c^

Note: Line item procedure values do not sum to total due to certain patients undergoing more than one subsequent surgical procedure on the same day.

a Amount censored due to count <11.

b All p-values have been adjusted by propensity score for age at index, gender, race, year of index, and all baseline comorbidities.

c Incidence of this subsequent intervention was statistically significantly lower for laminectomy patients.

### 3.3 Incidence of harms

*mild* patients experienced harms at a rate of 1.9 % compared to 5.8 % for outpatient laminectomy (p < 0.0001). Laminectomy patients experienced wound problems at a rate of 3.1 % versus 1.0 % for *mild* (p < 0.0001), and life-threatening complications at a rate of 2.1 % versus 1.0 % for *mild* (p < 0.0001). Injuries to the spinal cord, nerve roots or dura were reported at a rate of 1.4 % for laminectomy compared to no reported injuries for *mild* patients. Deep vein thrombosis was experienced by less than 11 laminectomy patients (exact number censored) and by none of the *mild* patients. All-cause mortality within 30 days of index occurred in 0.2 % of laminectomy (14 patients) and in less than 11 *mild* (exact number censored). (See Table 5.).

**Table 5:** Incidence of harms during two-year follow-up.

Harms	*mild* N = 2197		Outpatient Laminectomy N = 7416		p-value^a^
Incidence of any harm	41	1.9 %	433	5.8 %	<0.0001^b^
Wound complications	22	1.0 %	231	3.1 %	<0.0001^b^
Life-threatening complications^c^	22	1.0 %	155	2.1 %	<0.0001^b^
Acute MI	11	0.5 %	79	1.1 %	0.0004^b^
Pneumonia	d	d	51	0.7 %	n/a
Respiratory problems	d	d	49	0.7 %	n/a
Stroke	d	d	20	0.3 %	n/a
Pulmonary embolism	d	d	17	0.2 %	n/a
Spinal cord or nerve root injury, and dural tear	0	0 %	107	1.4 %	n/a
DVT	0	0 %	d	d	n/a
Heterotopic ossification	0	0 %	d	d	n/a
Death	d	d	14	0.2 %	n/a

a All p-values have been adjusted by propensity score for age at index, gender, race, year of index, and all baseline comorbidities.

b Incidence of this harm was statistically significantly lower for *mild* patients.

c Includes acute myocardial infarction, pneumonia, respiratory problems, stroke and pulmonary embolism.

d Amount censored due to count <11.

### 3.4 Overall composite rate of subsequent surgical procedures and harms

Combined rate of overall subsequent surgical procedures and harms was similar between groups at 10.8 % for *mild* and 11.0 % for outpatient laminectomy (p = 0.3845). (See Table 6.).

**Table 6:** Overall rate of subsequent surgical procedures and harms during two-year follow-up.

	*mild* N = 2197		Outpatient Laminectomy N = 7416		p-value^a^
Subsequent surgical procedure rate	198	9.0 %	410	5.5 %	<0.0001^b^
Incidence of any harm	41	1.9 %	433	5.8 %	<0.0001^c^
Composite rate of harms and subsequent surgical procedures	237	10.8 %	818	11.0 %	0.3845

a All p-values have been adjusted by propensity score for age at index, gender, race, year of index, and all baseline comorbidities.

b Rate was statistically significantly lower for laminectomy patients.

c Incidence was statistically significantly lower for *mild* patients.

## 4 Discussion

*mild* patients in this study were older and presented with more comorbidities than outpatient laminectomy patients. This may reflect a preference by providers to utilize *mild* for patients with more comorbidities or advanced age, and for whom open procedures are either not indicated or not desired [[3], [4], [5], [6]].

Subsequent surgical procedures were confirmed to involve the lumbar region, but it was not possible to determine if subsequent surgical procedures were performed at the same spinal level as the index procedure. This method for identifying subsequent surgical procedures has been documented in numerous previous reports analyzing surgical spine procedures using administrative claims [4,[10], [11], [12], [13], [14], [15]]. A review of these publications indicates a range of two-year subsequent surgical procedure rates for open decompression patients ranging from 7.2 % to just over 10 %. The subsequent surgical procedure rate for outpatient laminectomy patients in this report was 5.5 %, which is somewhat lower than these rates. This may reflect the requirement in this study for subsequent surgical procedures to include an LSS with NC diagnosis code in the claim's primary position. *mild* patients reported a 9.0 % rate of subsequent surgical procedures. While subsequent surgical procedure rates have not been previously reported for *mild* using administrative claims data, there have been two Level 1 RCTs with two-year follow-up that can be used as benchmarks. The ENCORE study investigators reported a two-year 5.6 % subsequent surgical procedure rate at the index level for *mild* patients [7]. In the MOTION RCT, 3.1 % of *mild* patients received a subsequent surgical procedure at the index level through two years [17]. These *mild* subsequent surgical procedure rates of 3.1 % and 5.6 % at two years are lower than the 9.0 % reported in this study, which likely reflects overcounting in Medicare claims data since repeat surgery cannot be confirmed to occur at the index level, but only in the overall lumbar region. Given this, the outpatient laminectomy subsequent surgical procedure rate may be overstated as well. In any event, the comparison of these two cohorts uses consistent coding rules and therefore provides valid relative subsequent surgical procedure outcomes.

At 1.9 %, the incidence of harms experienced by *mild* patients was statistically significantly lower than the 5.8 % reported by laminectomy patients. Specifically, outpatient laminectomy patients experienced wound problems at a rate three times higher, and life-threatening complications at a rate two times higher than *mild* patients, with both differences reaching statistical significance. Life threatening complications included acute myocardial infarction, pneumonia, respiratory problems, pulmonary embolism, and stroke. The only sub-category of life-threatening complications that included 11 or more patients in both cohorts and therefore was not censored, was acute myocardial infarction which occurred at a 0.5 % rate in the *mild* group and a rate over two times higher (1.1 %) for laminectomy patients (p = 0.0004). Reports of the ENCORE RCT at two-year follow-up indicated a 1.3 % rate of device- or procedure-related adverse events for *mild* patients with no serious device- or procedure-related adverse events occurring in the study [7]. In the MOTION RCT, through two years there were no reports of device- or procedure-related adverse events for *mild* patients [17]. These adverse event rates for *mild* in two Level 1 RCTs are in line with the 1.9 % reported in this analysis of Medicare claims. As a benchmark for open decompression at two years, the SPORT study reported an intraoperative complication rate of 10 %, and an overall postoperative complication rate of 12 % [18]. These SPORT complication rates are higher than the 5.8 % reported here for outpatient laminectomy and may reflect improvements in surgical techniques or the push toward outpatient surgery that has occurred since publication of the SPORT study.

This analysis compared outcomes of patients treated with *mild* to those who underwent outpatient laminectomy. Claims data showed that 20 % of ALL laminectomy patients were treated in the inpatient setting, and because of that sizeable percentage, a supplementary comparison of *mild* versus ALL laminectomy patients was conducted. The rate of subsequent surgical procedures for ALL laminectomy patients was 5.4 % which was statistically significantly lower than 9.0 % for *mild*. The incidence of any harm for ALL laminectomy patients was 8.4 % which was statistically significantly higher than 1.9 % for *mild*. It is interesting to note the differences in laminectomy patient two-year outcomes when comparing ALL laminectomy patients to the outpatient laminectomy cohort used in this study. The subsequent surgical procedure rate was similar between ALL laminectomy patients and the subgroup containing only outpatient laminectomy patients (5.4 % and 5.5 %, respectively). The incidence of harms was higher for ALL laminectomy patients at 8.4 % versus 5.8 % for outpatient laminectomy only, reflecting an incidence of harms for inpatient laminectomy patients of 18.8 %. The higher rate of harms for inpatient laminectomy may be attributable to inpatient laminectomy patients being older than outpatient laminectomy patients (74.5 years vs. 73.4 years, respectively; p < 0.0001) and having more baseline comorbidities (mean of 5.6 vs. 4.8, respectively; p < 0.0001).

In a meta-analysis of published studies of surgical treatment for LSS, Bays and colleagues reported that higher levels of presenting comorbidities were associated with an increased risk for complications related to the index surgical procedure [19]. In this study, despite the *mild* cohort having a significantly higher level of baseline comorbidities, incidence of harms for *mild* was significantly less, suggesting lower procedural risk for the minimally invasive *mild* procedure in higher risk patients.

This study used real-world Medicare claims data to compare outcomes for two treatments that occur at distinctly different timepoints on the LSS treatment algorithm. *mild* has been recommended by multiple published studies and reviews for use either as soon as LSS is diagnosed or after failure of the first ESI [3,[6], [7], [8], [9]]. Conversely, laminectomy, which involves longer recovery times and higher complication rates, appears later in the treatment continuum. Consistent with the marked separation in treatment algorithm positioning, these procedures are under the purview of different physician specialties with *mild* most often performed by interventional pain management, anesthesiology, physical medicine and rehabilitation, and interventional radiology physicians, while laminectomy is performed by spine surgeons. With different treating specialties and patients at divergent positions in their personal treatment journeys, use of Medicare claims data offers a valuable insight into real-world, unbiased comparison of patient outcomes. Availability of large numbers of patients combined with use of propensity scoring allowed for a balanced and valid method of comparing two procedures that otherwise would be impossible to reasonably study in a traditional controlled clinical trial setting.

Population-based studies generally include a large number of patients and therefore have strong statistical power. Specifically, this study used CMS RIF files which include all medical claims for 100 % of Medicare beneficiaries and are the most complete data source for Medicare claims analyses. There are some important limitations to this study. Using Medicare claims, it is unknown if subsequent surgical procedures involve the same spinal level as index, likely resulting in overcounting. Degree and anatomical cause of stenosis and number of spinal levels are not defined using claims data, and common patient reported outcome measures are not available. The observational nature of population-based studies can limit selection criteria precision potentially resulting in some residual confounding, although propensity scoring was used to reduce this possibility. Analysis of outcomes with patient counts less than 11 could not be performed due to required censoring [10,13,20].

## 5 Conclusion

*mild* can be considered a viable option for treatment of LSS with NC as evidenced by real-world data in this study. At two-years, *mild* patients experienced fewer harms and underwent more subsequent surgical procedures than outpatient laminectomy patients. The higher rate of subsequent surgical procedures for *mild* may be attributable to its position earlier in the LSS treatment algorithm. The overall rate of harms and subsequent surgical procedures was similar between groups, suggesting that *mild* should be considered as a treatment option, particularly for older patients with multiple comorbidities.

## Declaration of competing interest

PS reports consulting or research grants from Medtronic, Saluda Medical, Biotronik, AIS Healthcare, Nalu, SPR Therapeutics and funded research from Vertos Medical. MD is a consultant for Abbott, Biotronik, Camber Spine, Globus, Nevro and LifeSpine. DR and NS report no conflicts. LP is a consultant for Medtronic and Saluda Medical, and holds stock options for Nalu and Saluda. JH reports no conflicts.
